# Evaluation of Machine Learning Models for Predicting Antimicrobial Resistance of *Actinobacillus pleuropneumoniae* From Whole Genome Sequences

**DOI:** 10.3389/fmicb.2020.00048

**Published:** 2020-02-06

**Authors:** Zhichang Liu, Dun Deng, Huijie Lu, Jian Sun, Luchao Lv, Shuhong Li, Guanghui Peng, Xianyong Ma, Jiazhou Li, Zhenming Li, Ting Rong, Gang Wang

**Affiliations:** ^1^Institute of Animal Science, Guangdong Academy of Agricultural Sciences, Guangzhou, China; ^2^State Key Laboratory of Livestock and Poultry Breeding, Guangzhou, China; ^3^Key Laboratory of Animal Nutrition and Feed Science of Ministry of Agriculture (South China), Guangzhou, China; ^4^Guangdong Engineering Technology Research Center of Animal Meat Quality and Safety Control and Evaluation, Guangzhou, China; ^5^National Veterinary Microbiological Drug Resistance Risk Assessment Laboratory, College of Veterinary Medicine, South China Agricultural University, Guangzhou, China

**Keywords:** machine learning, Support Vector Machine, Set Covering Machine, antimicrobial resistance, *Actinobacillus pleuropneumoniae*, genomics

## Abstract

Antimicrobial resistance (AMR) is becoming a huge problem in countries all over the world, and new approaches to identifying strains resistant or susceptible to certain antibiotics are essential in fighting against antibiotic-resistant pathogens. Genotype-based machine learning methods showed great promise as a diagnostic tool, due to the increasing availability of genomic datasets and AST phenotypes. In this article, Support Vector Machine (SVM) and Set Covering Machine (SCM) models were used to learn and predict the resistance of the five drugs (Tetracycline, Ampicillin, Sulfisoxazole, Trimethoprim, and Enrofloxacin). The SVM model used the number of co-occurring k-mers between the genome of the isolates and the reference genes to learn and predict the phenotypes of the bacteria to a specific antimicrobial, while the SCM model uses a greedy approach to construct conjunction or disjunction of Boolean functions to find the most concise set of k-mers that allows for accurate prediction of the phenotype. Five-fold cross-validation was performed on the training set of the SVM and SCM model to select the best hyperparameter values to avoid model overfitting. The training accuracy (mean cross-validation score) and the testing accuracy of SVM and SCM models of five drugs were above 90% regardless of the resistant mechanism of which were acquired resistant or point mutation in the chromosome. The results of correlation between the phenotype and the model predictions of the five drugs indicated that both SVM and SCM models could significantly classify the resistant isolates from the sensitive isolates of the bacteria (*p* < 0.01), and would be used as potential tools in antimicrobial resistance surveillance and clinical diagnosis in veterinary medicine.

## Introduction

Antimicrobial resistance (AMR) in bacteria from humans and food-producing animals is becoming an urgent threat to the control of bacterial infections. Identification of strains resistant or susceptible to certain antibiotics is essential in fighting against antibiotic-resistant pathogens. Typically, the determination of antimicrobial susceptibility is done either by disk diffusion or minimum inhibitory concentration (MIC) assays. Identification of resistance-specific markers by PCR or microarray hybridization not only corroborates phenotypic results but is also useful for epidemiological purposes, as there are often multiple different genes that can confer resistance to a given antimicrobial agent (Bossé et al., 2017). With the increasing throughput and decreasing cost of DNA sequencing, whole genome sequencing (WGS) may be an alternative for routine surveillance of resistance profiles and for identification of emerging resistances (Mahé and Tournoud, 2018).

*Actinobacillus pleuropneumoniae* causes porcine pleuropneumonia, which is present in almost all the countries of the world. Pleuropneumonia can affect all ages of pigs and may result in great economic losses in pig production particularly as it causes serious respiratory distress and death. *A. pleuropneumoniae* is divided into 15 serotypes based on the antigenic properties of capsular polysaccharides and cell wall lipopolysaccharides. None of the serotype provides a cross-immune response for another serotype and therefore restricts the application of vaccine (Kim et al., 2016). *A. pleuropneumoniae* can be killed by using effective antimicrobials. However, resistant mutants increased gradually due to the misuse of antimicrobials (Zhang et al., 2018). Knowledge of resistance profiles for *A. pleuropneumoniae* is required to inform treatment decisions.

The presence or absence of specific resistance genes must be associated with resistance (and susceptibility) to particular antibiotics, and then the resistance profiles for all genes in a particular isolate must be added together to provide the predicted susceptibility profile for that organism. The routine studies make genotype-to-phenotype predictions based on identifying the AMR genes in the draft genomes via web servers like ResFinder (Zankari et al., 2012), the Comprehensive Antibiotic Resistance Database (CARD) (McArthur et al., 2013), and Resfams (Gibson et al., 2015).

With the help of computational tools, reference-based or reference-free machine-learning algorithms have been used increasingly to build models that correlate genomic variations with phenotypes. In supervised learning, each example consists of an input and an expected outcome. The goal of the algorithm is to learn a model that accurately maps any input to the correct outcome.

In this study, we propose to apply the Support Vector Machine (SVM) and Set Covering Machine (SCM) algorithm to accurately predict their phenotype against five antimicrobial agents (Tetracycline, Ampicillin, Sulfisoxazole, Trimethoprim, and Enrofloxacin) from the whole genomes of 96 isolates of *A. pleuropneumoniae*.

## Materials and Methods

### Data

The WGS reads and binary resistance phenotypes of 5 antimicrobial agents (tetracycline, ampicillin, sulfisoxazole, trimethoprim, and enrofloxacin) of 96 isolated strains of *A. pleuropneumoniae* data were obtained from Bossé et al. (2017). The WGS reads were downloaded from the European Nucleotide Archive (Study: PRJEB2343^1^) and the phenotypes of the isolates against the antimicrobial agents were downloaded from the Supplementary Material of the same study^2^. Acquired resistance genes of the antimicrobial agents were downloaded from ResFinder Database as reference genes^3^.

For enrofloxacin, even though resistance might be mediated by the acquired *qnr* genes, resistance to fluoroquinolones in the *A. pleuropneumoniae* is most often mediated by mutations in the target genes *gyrA*, *parC*, and *parE* (Wang et al., 2010; Pesesky et al., 2016; Zhang et al., 2018). Therefore, gene sequences of the quinolone resistance determining regions (QRDR) of *gyrA* (residues 68–106), *parC* (residues 68–106), and *parE* (residue 425–478) of all the isolates were translated into amino acid and aligned with the same regions of the reference *gyrA* (GenBank accession number ABN73394), *parC* (GenBank accession number ABN73680) and *parE* (GenBank accession number ABN74341), respectively. All the DNA sequences of QRDR with no mutation in amino acid were appended into a FASTA file as reference genes (see Supplementary Material).

The WGS reads were further assembled using Velvet 1.2.08 (Zerbino and Birney, 2008). The contigs of the strains along with the AMR genes downloaded from ResFinder Database and the gene sequences of QRDR for recognition of enrofloxacin point mutation were subsequently split into k-mers (sequence of k nucleotides) of length 31 using the Ray Surveyor tool (Déraspe et al., 2017).

### Reference-Based SVM Model

With the input of resistance genes of interest as reference genes, the matrix of the co-occurring k-mers in the genome of the strains and the reference genes were simultaneously built by the Ray Surveyor tool during the splitting process. Support Vector Machine (SVM; radial basis function kernel) used the number of co-occurring k-mers of the strain and the reference genes of the specific antimicrobial to learn and predict the phenotypes of each isolate. The SVM was implemented in the Python sklearn package^4^.

The dataset was randomly divided into three subsets of equal size by the ID of the strains, and two subsets were used for training while the other one was used for testing. The training and testing process repeated three times so that every subset of the strains could be used to evaluate the performance of the model.

### Reference-Free SCM Model

Unlike the SVM model which included k-mers of reference genes in the dataset, the SCM used to learn sparse and interpretable models of phenotypes by reference-free k-mers comparisons are performed implemented in Kover, an open-source software implemented in the Python and C programming languages^5^. Kover automates the machine learning analysis (e.g., dataset splitting, model selection, and model evaluation) without making assumptions about the underlying genetic mechanisms. The k-mers and phenotypic data of all the strains were used and packaged into a Kover dataset, and then split the dataset into a training set (2/3 of the Kover data) and a testing set (1/3 of the Kover data) according to the same ID of the datasets of the SVM model. The training set was used to learn a model containing combination rules of both conjunction (logical-AND) and disjunction (logical-OR) at most 5 rules, the testing dataset was used for testing the accuracy of the model.

### Model Selection and Performance Evaluation

In order to minimize the waste of the training dataset and avoid overfitting, five-fold cross-validation was performed on the training set of the SVM and SCM model to select the best hyperparameter values. The best hyperparameter values selected from the five-folds cross-validation were then averaged and chosen to evaluate the performance of the model.

The performances of the SVM and SCM model were evaluated in terms of sensitivity, specificity, accuracy, and precision. They were defined as: sensitivity = TP/(TP + FN), specificity = TN/(TN + FP), accuracy = (TP + TN)/(TP + FP + TN + FN), and precision = TP/ (TP + FP). Where TP was the number of resistant strains predicted to be resistant, TN was the number of sensitive strains predicted to be sensitive, FP was the number of sensitive strains predicted to be resistant, and FN was the number of resistant strains predicted to be sensitive.

## Results

**FIGURE 1:**
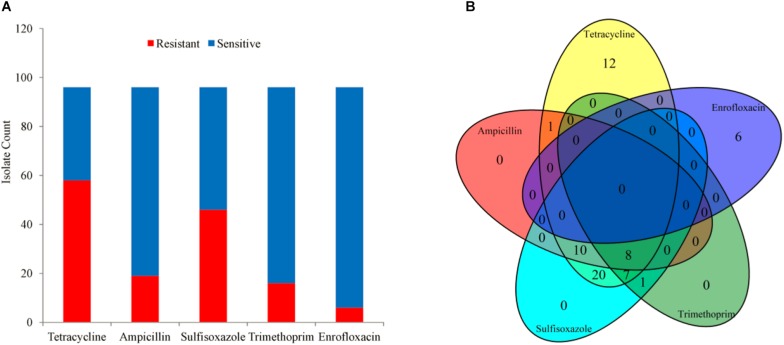
The phenotype of 96 isolates. **(A)** Bar plot of phenotype availability for the different drugs. **(B)** Venn diagram quantifying the number of instances of co-occurrence of resistance between drugs.

A total of 96 clinical *A. pleuropneumoniae* isolates were included in the study, with 58, 19, 46, 16, 6 of the isolates resistant to Tetracycline, Ampicillin, Sulfisoxazole, Trimethoprim, and Enrofloxacin, respectively. There were 8 isolates were resistant to four kinds of antimicrobials (Tetracycline, Ampicillin, Sulfisoxazole, and Trimethoprim); 17 isolates were resistant to 3 kinds of antimicrobials, 10 of them were resistant to Tetracycline, Ampicillin, and Sulfisoxazole, 7 of them were resistant to Tetracycline, Ampicillin, and Trimethoprim, respective; 22 isolates were resistant to 2 kinds of antimicrobials, 20 of them were resistant to Tetracycline and Sulfisoxazole, one of them was resistant to Tetracycline and Ampicillin, one of them was resistant to Sulfisoxazole and Trimethoprim, respectively; 18 isolates were resistant to single antimicrobial, 12 and 6 of them were resistant to Tetracycline and Enrofloxacin, respectively; and 31 were sensitive to all kinds of the five antimicrobials (Figure 1).

The *gyrA* QRDR DNA fragments of all the 90 Enrofloxacin sensitive isolates were the same as that region of the reference *gyrA*, while part of the isolates contained the same QRDR DNA fragments as the reference *parC* or *parE* genes. And including those fragments, there were 5 and 2 DNA fragments in the 90 Enrofloxacin sensitive isolates that code the same amino acid as reference *parC* and *parE*, respectively.

**FIGURE 2:**
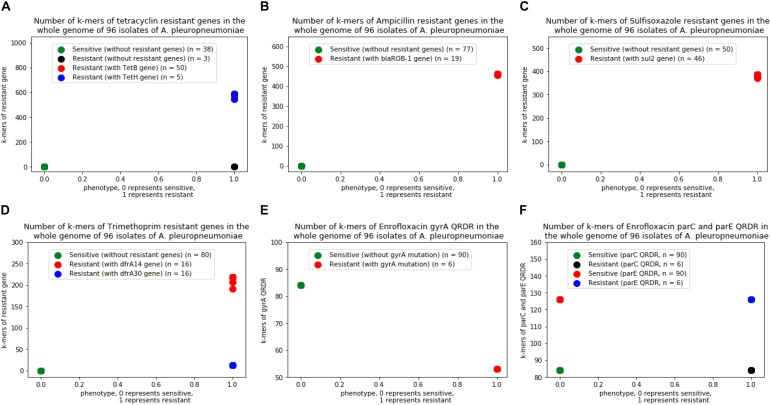
Number of k-mers of resistant genes in the whole genome of 96 isolates of *A. pleuropneumoniae*. **(A)** Isolates with k-mers of *tetB* and *tetH* genes, **(B)** Isolates with k-mers of blaROB-1 gene, **(C)** Isolates with k-mer of sul2 gene, **(D)** Isolates with k-mers of dfrA14 and dfrA30 genes, **(E)** Isolates with k-mers in gene of gyrA QRDR without point mutation, **(F)** Isolates with k-mers in genes of parC and parE QRDR without point mutation.

A total of 4,299,871 distinct k-mers of length 31 were obtained from the 96 genomes of *A. pleuropneumoniae*. By comparing the k-mers of the genes downloaded from ResFinder, a range of 509∼607 k-mers of *tet*(B) gene were found in the genome of 50 strains, 540∼613 k-mers of *tet*(H) gene in 5 strains, 454∼463 k-mers of *blaROB-1* gene in 19 strains, 299∼402 k-mers of *sul2* gene in 46 strains, and 172∼236 k-mers of *dfrA14* gene together with 13 k-mers of *dfrA30* gene in 16 strains of the bacteria, respectively. For enrofloxacin, 53 and 84 k-mers of *gyrA* QRDR in the genomes of 7 and 89 isolates, 84 and 126 k-mers of *parC* and *parE* QRDR in 96 isolates, respectively (Figure 2). No k-mer of *qnr* genes were found in the genomes of the isolates.

**FIGURE 3:**
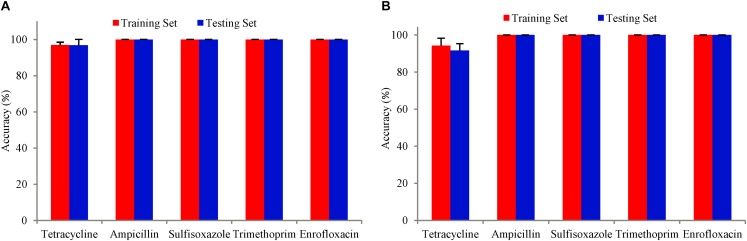
Bars with red color show the mean accuracy for the tuned model with five-fold cross-validation on the training dataset. Bars with blue color are the accuracy of the tuned model on the test dataset. The error bars are standard deviations. **(A)** SVM model, **(B)** SCM model.

The training accuracy (mean cross-validation score) and the testing accuracy of SVM and SCM models of five drugs were above 90% (Figure 3), indicating that both of the SVM and SCM models were not overfitted. Average and standard deviation of the sensitivity, specificity, accuracy, and precision measured on the 3 randomly partition testing sets representing the whole 96 unduplicated strains of the bacteria were provided in Table 1. The accuracies of Ampicillin, Sulfisoxazole, Trimethoprim, and Enrofloxacin were 1.00 ± 0.00, indicating that no false positive and no false-negative strain of bacteria were predicted by both of the models.

**TABLE 1:** Prediction metrics on test datasets using the best performing SVM and SCM models.

	**SVM**				**SCM**			
	**Sensitivity**	**Specificity**	**Accuracy**	**Precision**	**Sensitivity**	**Specificity**	**Accuracy**	**Precision**
Tetracycline	0.95 ± 0.05	1.00 ± 0.00	0.97 ± 0.03	1.00 ± 0.00	0.86 ± 0.08	1.00 ± 0.00	0.92 ± 0.04	1.00 ± 0.00
Ampicillin	1.00 ± 0.00	1.00 ± 0.00	1.00 ± 0.00	1.00 ± 0.00	1.00 ± 0.00	1.00 ± 0.00	1.00 ± 0.00	1.00 ± 0.00
Sulfisoxazole	1.00 ± 0.00	1.00 ± 0.00	1.00 ± 0.00	1.00 ± 0.00	1.00 ± 0.00	1.00 ± 0.00	1.00 ± 0.00	1.00 ± 0.00
Trimethoprim	1.00 ± 0.00	1.00 ± 0.00	1.00 ± 0.00	1.00 ± 0.00	1.00 ± 0.00	1.00 ± 0.00	1.00 ± 0.00	1.00 ± 0.00
Enrofloxacin	1.00 ± 0.00	1.00 ± 0.00	1.00 ± 0.00	1.00 ± 0.00	1.00 ± 0.00	1.00 ± 0.00	1.00 ± 0.00	1.00 ± 0.00

Even though 3 and 8 out of 58 phenotype resistant strains were predicted to be sensitive for tetracycline from SVM and SCM model, respectively, the sensitivity and accuracy of both of the models were still high enough for prediction. Of the 3 false-negative isolates (MIDG3342, MIDG3352, and MIDG3356) predicted by SVM and SCM, no acquired tetracycline-resistant genes were found in the genome of those isolates. Of the other 5 false negative isolates predicted by SCM, all of them were found to carry the *tetH* gene and predicted to be true positive by SVM.

Correlations between the phenotype and the model predictions of 3 subsets of testing datasets represented 96 unduplicated strains of *A. pleuropneumoniae* isolates were shown in Table 2. The results indicated that both SVM and SCM models could significantly classify the resistant isolates from the sensitive isolates of the bacteria (*p* < 0.01).

**TABLE 2:** Correlation of phenotype and model predictions of SVM and SCM models.

**Antimicrobial agent**	**Number of isolates classified by**	**SVM**		**SCM**	
	**phenotype (R, S)**	**Number of isolates predicted to be TP and TN (TP, TN)**	**Correlation of phenotype to the model prediction**	**Number of isolates predicted to be TP and TN (TP, TN)**	**Correlation of phenotype to the model prediction**
Tetracycline	(58, 38)	(55, 38)	*p* < 2.2e−16	(50, 38)	*p* < 2.2e−16
Ampicillin	(19, 77)	(19, 77)	*p* < 2.2e−16	(19, 77)	*p* < 2.2e−16
Sulfisoxazole	(46, 50)	(46, 50)	*p* < 2.2e−16	(46, 50)	*p* < 2.2e−16
Trimethoprim	(16, 80)	(16, 80)	*p* < 2.3e−16	(16, 80)	*p* < 2.3e−16
Enrofloxacin	(6, 90)	(6, 90)	*p* < 1.1e−09	(6, 90)	*p* < 1.1e−09

*R resistant, S sensitive. Correlation between phenotype and model predictions were calculated using Fisher’s exact test in python.*

## Discussion

Support Vector Machine (SVM) has been applied to several biological problems such as prediction of protein-protein interactions, homology detection, gene expression analysis, drug discovery, and drug resistance analysis (Cui et al., 2012; Li et al., 2016; Kouchaki et al., 2018). To our knowledge, it’s the first time to use the SVM model to predict the drug resistance based on the counts of co-occurring k-mers between the genome and the reference resistance genes. Using the reference gene fragments of QRDR built by the authors could cover the deficit that no point mutation databases provide the reference genes specific for *A. pleuropneumoniae*. And this new method could be used for phenotype prediction of the other genes or bacteria with point mutation.

Unlike the KmerResistance, which uses the “winner takes all strategy”(Clausen et al., 2016), the exact number of co-occurring k-mers between the genome and the reference resistance genes were counted. The supervised machine learning itself can learn from the situation where k-mers not being able to match due to the mismatch, indel, non-perfect assembly, or genomic rearrangements in the query genome from the training dataset and predict the correct answer while the same situation happened in the test dataset.

The Set Covering Problem is a classical question in combinatorics, computer science, operations research, and complexity theory. As of now, one of the most relevant applications of SCP is given by crew scheduling problems in railway and mass-transit transportation companies, where a given set of trips has to be covered by a minimum-cost set of pairings (Caprara et al., 2000). In this study, The SCM algorithm uses a greedy approach to construct conjunction (logical-AND) or disjunction (logical-OR) of Boolean functions to find the most concise set of genomic features (k-mers) that allows for accurate prediction of the phenotype. A conjunction model assigns the positive class to a genome if all the rules output true, whereas a disjunction model does the same if at least one rule outputs true. The method was validated by generating models that predict the antibiotic resistance of *C. difficile*, *M. tuberculosis*, *P. aeruginosa*, and *S. pneumoniae* for 17 antibiotics (Drouin et al., 2016). The obtained models, implemented in Kover, were proven to be accurate, faithful to the biological pathways targeted by the antibiotics, and they provide insight into the process of resistance acquisition.

The numbers of isolates predicted to be sensitive or resistant by SVM was exactly the same as the result predicted by mapping the reference resistant genes against the assembly of the WGS data (Bossé et al., 2017). This indicated that the SVM model is excellent in classifying the phenotype of the bacteria. In general, reference-based SVM model should be equally successful whether they are applied to a small or large set of pathogens since the accuracy of the prediction rely mainly on whether there were reference resistant genes in the reference databases like ResFinder or other built-in databases.

Until now, no point mutation was reported in the amino acid of GyrB QRDR of *A. pleuropneumoniae*. Of the amino acid of *parE* QRDR (residues 440–479) of 96 isolates in this study, 29 sensitive isolates had substitutions of D479E in the *parE* protein. The other 61 sensitive isolates and the 6 resistant isolates did not have substitutions of D479E comparing with the amino acid of reference *parE* gene. The finding indicated that mutation of D479E in the *parE* gene might not be related to the resistant of the bacteria against Enrofloxacin. So, DNA sequences of QRDRs of *gyrA* (residues 68–106), *parC* (residues 68–106), and *parE* (residue 425–478) of sensitive isolates were chosen and appended to a FASTA file as reference genes for the SVM model to learn and predict the phenotypes of the bacteria against Enrofloxacin.

The SCM model, regardless of the resistant mechanism of which were acquired AMR genes or point mutation in the chromosome, by comparing the difference of the k-mers between the resistant strains and the sensitive strains, finds the most concise set of equivalent k-mers that allows for accurate prediction of the phenotype.

Any approach that uses machine learning models requires adequate input data to form a “training set” to train the machine learning model and a “testing set” to assess the performance of the model (Macesic et al., 2017). Among the five antimicrobial agents, the resistant background of *A. pleuropneumoniae* against tetracycline is more complicated than the others. There were 58 phenotype resistant strains, with 50 isolates carrying *tet*(B), 5 isolates carrying *tet*(H), and 3 isolates did not have any tetracycline resistance genes detected. And up to now, we still could not able to collect the whole genome of the *A. pleuropneumoniae* with *tet*(H) genes publicly, therefore, after randomly split the limited data into training set or testing set, the SCM model did not have enough sample to learn from the training dataset and therefore lead to a relative lower accuracy while predicting the testing set of the model.

Both models have advantages and shortcuts. The reference-based SVM model performs well at classifying resistance from sensitive isolates regardless of the sample size of the training set since the counts of co-occurring k-mers between the genome and the reference resistance genes of the resistant isolates are significantly different from that of the sensitive isolates (Figure 2). But this method relies mainly on the database and therefore cannot be used for predictions where resistance mechanisms have yet to be identified. The SCM model should need enough proportion of true phenotype data against false phenotype data as input to form a “training set” to train the model, but it provides a unique approach for deciphering, *de novo*, new biological mechanisms without the need for prior information (Drouin et al., 2016).

Even though both of the models can use raw reads to learn and predict the phenotype of the bacteria, it is recommended to use the assembled contigs as input data, since the genome assembly can increase the quality of the k-mer representation, reduces the number of unique k-mers and thus makes the process of splitting the genome into k-mers and building the matrix encoding the presence or absence of all k-mers by Ray Surveyor tool much faster (Drouin et al., 2016; Mahé and Tournoud, 2018).

## Data Availability Statement

The raw data supporting the conclusions of this article will be made available by the authors, without undue reservation, to any qualified researcher.

## Author Contributions

ZCL, TR, and GW designed the study and machine learning algorithm. DD, HL, JS, and LL analyzed the data and evaluated the biological relevance of the models. SL, GP, XM, JL, and ZML acquired the data and prepared it for analysis. ZCL wrote the manuscript.

## Conflict of Interest

The authors declare that the research was conducted in the absence of any commercial or financial relationships that could be construed as a potential conflict of interest.

## Footnotes

1 https://www.ebi.ac.uk/ena/data/view/PRJEB2343

2 https://www.frontiersin.org/article/10.3389/fmicb.2017.00311/full#supplementary-material

3 https://bitbucket.org/genomicepidemiology/resfinder_db/downloads/

4 http://scikit-learn.org/

5 https://github.com/aldro61/kover/
